# Supplementary material for: Kinetics of anti-SARS-CoV-2 antibodies and hematological parameters in hospitalized pre-vaccination COVID-19 patients in Peru
Source: PeerJ. 2025 Aug 22;13:e19771. doi: 10.7717/peerj.19771 (PMC12377358; doi:10.7717/peerj.19771)
Supplement: Supplemental Information 3 [file peerj-13-19771-s003.doc]

**STROBE Statement Checklist for cohort studies**

**Manuscript Title:** Kinetics of anti-SARS-CoV-2 antibodies and hematological parameters in hospitalized COVID-19 patients in Peru pre-vaccination.

This checklist has been prepared following the STROBE guidelines for reporting observational studies. The table below indicates where each required item is addressed within the manuscript. This checklist ensures compliance with the STROBE Statement standards for cohort studies, enhancing the transparency and completeness of the reporting.

|  | Item No | Recommendation | Checklist |  |
| --- | --- | --- | --- | --- |
| **Title and abstract** | 1 | (*a*) Indicate the study’s design with a commonly used term in the title or the abstract | Line 1-3 |  |
|  |  | (*b*) Provide in the abstract an informative and balanced summary of what was done and what was found | Line 34-55 |  |
| Introduction | | |  |  |
| Background/rationale | 2 | Explain the scientific background and rationale for the investigation being reported | Line 58-89 |  |
| Objectives | 3 | State specific objectives, including any prespecified hypotheses | Line 90-95 |  |
| Methods | | |  |  |
| Study design | 4 | Present key elements of study design early in the paper | Line 98 |  |
| Setting | 5 | Describe the setting, locations, and relevant dates, including periods of recruitment, exposure, follow-up, and data collection | Line 99-104 |  |
| Participants | 6 | (*a*) Give the eligibility criteria, and the sources and methods of selection of participants. Describe methods of follow-up | Line 105-108 |  |
|  |  | (*b*) For matched studies, give matching criteria and number of exposed and unexposed | Line 109-112 |  |
| Variables | 7 | Clearly define all outcomes, exposures, predictors, potential confounders, and effect modifiers. Give diagnostic criteria, if applicable | Line 114-139 |  |
| Data sources/ measurement | 8* | For each variable of interest, give sources of data and details of methods of assessment (measurement). Describe comparability of assessment methods if there is more than one group | Line 114-123 |  |
| Bias | 9 | Describe any efforts to address potential sources of bias | Line 168-172 |  |
| Study size | 10 | Explain how the study size was arrived at | Line 162-163 |  |
| Quantitative variables | 11 | Explain how quantitative variables were handled in the analyses. If applicable, describe which groupings were chosen and why | Line 141-150 |  |
| Statistical methods | 12 | (*a*) Describe all statistical methods, including those used to control for confounding | Line 141-161 |  |
|  |  | (*b*) Describe any methods used to examine subgroups and interactions | Line 146-150 |  |
|  |  | (*c*) Explain how missing data were addressed | Line 163-166 |  |
|  |  | (*d*) If applicable, explain how loss to follow-up was addressed | Line 163-166 |  |
|  |  | (*e*) Describe any sensitivity analyses | Line 159-161 |  |
| Results | | |  |  |
| Participants | 13* | (a) Report numbers of individuals at each stage of study—eg numbers potentially eligible, examined for eligibility, confirmed eligible, included in the study, completing follow-up, and analysed | Line 181-192 |  |
|  |  | (b) Give reasons for non-participation at each stage | NA |  |
|  |  | (c) Consider use of a flow diagram | NA |  |
| Descriptive data | 14* | (a) Give characteristics of study participants (eg demographic, clinical, social) and information on exposures and potential confounders | Line 183-187 |  |
|  |  | (b) Indicate number of participants with missing data for each variable of interest | Supplementary Table 1 |  |
|  |  | (c) Summarise follow-up time (eg, average and total amount) | Line 181-192 |  |
| Outcome data | 15* | Report numbers of outcome events or summary measures over time | Line 181-214 |  |
| Main results | 16 | (*a*) Give unadjusted estimates and, if applicable, confounder-adjusted estimates and their precision (eg, 95% confidence interval). Make clear which confounders were adjusted for and why they were included | Line 193-294 |  |
|  |  | (*b*) Report category boundaries when continuous variables were categorized | Line 193-294 |  |
|  |  | (*c*) If relevant, consider translating estimates of relative risk into absolute risk for a meaningful time period | NA |  |
| Other analyses | 17 | Report other analyses done—eg analyses of subgroups and interactions, and sensitivity analyses | Line 295-306 |  |
| Discussion | | |  |  |
| Key results | 18 | Summarise key results with reference to study objectives | Line 313-450 |  |
| Limitations | 19 | Discuss limitations of the study, taking into account sources of potential bias or imprecision. Discuss both direction and magnitude of any potential bias | Line 451-462 |  |
| Interpretation | 20 | Give a cautious overall interpretation of results considering objectives, limitations, multiplicity of analyses, results from similar studies, and other relevant evidence | Line 313-462 |  |
| Generalisability | 21 | Discuss the generalisability (external validity) of the study results | Line 463-469 |  |
| Other information | | |  |  |
| Funding | 22 | Give the source of funding and the role of the funders for the present study and, if applicable, for the original study on which the present article is based | Submitted online |  |

*Give information separately for exposed and unexposed groups.
