# Supplementary material for: Kinetics of anti-SARS-CoV-2 antibodies and hematological parameters in hospitalized pre-vaccination COVID-19 patients in Peru
Source: PeerJ. 2025 Aug 22;13:e19771. doi: 10.7717/peerj.19771 (PMC12377358; doi:10.7717/peerj.19771)
Supplement: Supplemental Information 4 [file peerj-13-19771-s004.docx]

**Supplementary Table 1.** Comparison of IgM and IgG antibody levels (anti-S1 and anti-RBD) and hematological parameter values in patients hospitalized with COVID-19, classified by disease severity (moderate ARDS, severe ARDS non-ICU, and severe ARDS ICU) and days of hospitalization.

| **Variable** | **1 – 4 th hospitalization day** | | | | | **5 – 10 th hospitalization day** | | | | | **11 or more hospitalization day** | | | | |
| --- | --- | --- | --- | --- | --- | --- | --- | --- | --- | --- | --- | --- | --- | --- | --- |
|  | **N** | **Moderate ARDS N = 14*^1^*** | **Severe ARDS non-ICU N = 31*^1^*** | **Severe ARDS ICU N = 14*^1^*** | ***p-value^2^*** | **N** | **Moderate ARDS N = 12*^1^*** | **Severe ARDS non-ICU N = 28*^1^*** | **Severe ARDS ICU N = 12*^1^*** | ***p-value^2^*** | **N** | **Moderate ARDS N = 7*^1^*** | **Severe ARDS non-ICU N = 20*^1^*** | **Severe ARDS ICU N = 19*^1^*** | ***p-value^2^*** |
| **IgM anti-S1** | 59 | 0.2 (0.3) | 0.1 (0.2) | 0.1 (0.1) | 0.038 | 52 | 0.2 (0.4) | 0.2 (0.4) | 0.2 (0.2) | 0.6 | 46 | 0.2 (0.1) | 0.2 (0.2) | 0.1 (0.3) | 0.2 |
| **IgG anti-S1** | 59 | 1.4 (1.8) | 0.8 (1.3) | 0.7 (0.6) | 0.2 | 52 | 2.9 (1.0) | 2.1 (1.4) | 1.5 (0.8) | 0.022 | 46 | 3.3 (0.4) | 2.5 (0.5) | 2.6 (0.6) | 0.004 |
| **IgM anti-RBD** | 59 | 0.2 (0.2) | 0.2 (0.4) | 0.2 (0.1) | 0.9 | 52 | 0.3 (0.3) | 0.3 (0.3) | 0.3 (0.3) | 0.9 | 46 | 0.1 (0.0) | 0.4 (0.3) | 0.2 (0.2) | 0.001 |
| **IgG anti-RBD** | 59 | 1.5 (1.2) | 0.8 (0.9) | 0.7 (0.5) | 0.008 | 52 | 2.4 (0.7) | 1.7 (1.5) | 1.3 (0.4) | 0.017 | 46 | 2.9 (0.3) | 2.1 (0.5) | 2.2 (0.7) | 0.006 |
| **Hemoglobin** | 50 | 14.1 (1.8) | 14.6 (1.9) | 13.4 (2.3) | 0.5 | 51 | 14.3 (1.5) | 14.3 (2.0) | 13.1 (2.0) | 0.10 | 46 | 13.7 (1.3) | 14.4 (1.3) | 11.7 (2.3) | 0.001 |
| **Leukocytes** | 49 | 10.1 (5.6) | 10.4 (8.9) | 15.1 (8.2) | 0.10 | 51 | 6.1 (2.8) | 11.7 (5.1) | 10.2 (5.2) | 0.012 | 46 | 6.3 (0.9) | 7.4 (4.5) | 12.0 (3.5) | <0.001 |
| **Lymphocytes** | 50 | 6.0 (12.5) | 9.0 (6.0) | 5.0 (4.0) | 0.081 | 51 | 18.0 (9.0) | 8.0 (6.0) | 6.0 (1.5) | 0.006 | 46 | 18.0 (10.1) | 18.5 (10.8) | 9.0 (5.2) | <0.001 |
| **Absolute Lymphocytes** | 49 | 0.7 (0.4) | 0.9 (0.5) | 0.6 (0.5) | 0.5 | 50 | 1.2 (0.3) | 0.9 (0.6) | 0.6 (0.2) | 0.012 | 46 | 1.2 (0.6) | 1.4 (0.7) | 0.9 (0.4) | 0.005 |
| **Segmented Neutrophils** | 50 | 84.0 (12.8) | 84.0 (7.0) | 90.0 (5.0) | 0.086 | 51 | 77.0 (14.9) | 85.0 (8.3) | 89.0 (2.8) | 0.008 | 46 | 75.0 (11.6) | 74.5 (11.8) | 86.0 (12.0) | <0.001 |
| **Absolute Neutrophils** | 49 | 9.0 (7.3) | 8.9 (8.5) | 13.1 (9.1) | 0.087 | 50 | 4.5 (1.6) | 9.4 (5.8) | 9.3 (4.7) | <0.001 | 46 | 4.6 (1.6) | 5.3 (3.4) | 10.4 (4.3) | <0.001 |
| **Platelets** | 48 | 339,500.0 (133,250.0) | 338,000.0 (100,000.0) | 300,000.0 (104,500.0) | 0.5 | 49 | 468,000.0 (221,000.0) | 407,000.0 (162,500.0) | 268,000.0 (90,500.0) | 0.004 | 39 | 285,000.0 (63,000.0) | 437,500.0 (248,750.0) | 269,000.0 (119,250.0) | 0.002 |
| **CRP** | 46 | 94.0 (175.4) | 61.3 (63.5) | 145.0 (119.6) | 0.2 | 44 | 9.4 (37.5) | 52.2 (115.6) | 171.3 (217.5) | <0.001 | 33 | 35.3 (11.8) | 13.6 (30.0) | 126.8 (171.5) | <0.001 |
| **D-dimer** | 40 | 0.8 (0.5) | 0.5 (0.3) | 1.5 (1.7) | 0.087 | 41 | 0.8 (1.0) | 0.6 (0.5) | 3.5 (2.5) | 0.017 | 27 | 0.4 (0.0) | 0.7 (0.3) | 4.6 (2.4) | 0.001 |
| **Ferritin** | 55 | 595.1 (309.8) | 927.7 (857.2) | 892.5 (594.8) | 0.2 | 51 | 551.2 (297.2) | 1,220.0 (686.1) | 1,163.3 (620.9) | 0.005 | 43 | 305.1 (246.2) | 598.1 (236.8) | 1,036.6 (652.3) | <0.001 |
| **Creatinine** | 48 | 0.7 (0.2) | 0.7 (0.1) | 0.9 (0.5) | 0.2 | 51 | 0.7 (0.2) | 0.8 (0.2) | 0.8 (1.1) | 0.077 | 45 | 0.9 (0.2) | 0.8 (0.2) | 1.1 (2.1) | 0.2 |
| **aPTT** | 45 | 32.9 (3.0) | 29.6 (2.5) | 36.4 (6.9) | 0.020 | 47 | 34.6 (8.1) | 30.8 (4.8) | 35.8 (10.6) | 0.10 | 37 | 38.6 (5.2) | 32.7 (5.0) | 36.6 (7.9) | 0.018 |
| **Prothrombin Time** | 45 | 11.5 (1.2) | 11.2 (0.9) | 12.1 (0.8) | 0.053 | 47 | 11.5 (0.6) | 11.5 (1.2) | 11.6 (2.0) | >0.9 | 37 | 11.6 (0.3) | 11.4 (1.2) | 11.2 (0.5) | 0.15 |
| **SpO_2_/FiO_2_** | 56 | 240.0 (139.5) | 153.9 (144.9) | 180.5 (140.4) | 0.6 | 51 | 300.0 (163.0) | 237.5 (152.9) | 161.9 (113.6) | 0.020 | 46 | 303.1 (189.8) | 317.9 (106.3) | 170.0 (71.3) | <0.001 |
| **NLR** | 49 | 14.9 (14.9) | 9.4 (8.4) | 24.3 (17.9) | 0.065 | 50 | 3.9 (2.6) | 10.2 (8.1) | 15.0 (4.6) | 0.002 | 46 | 4.2 (3.0) | 4.1 (3.9) | 8.9 (9.0) | <0.001 |
| *^1^* Median (IQR) | | | | | | | | | | | | | | | |
| *^2^* Kruskal-Wallis rank sum test | | | | | | | | | | | | | | | |
